# Effectiveness of a telerehabilitation intervention using ReCOVery APP of long COVID patients: a randomized, 3-month follow-up clinical trial

**DOI:** 10.1038/s41598-023-35058-y

**Published:** 2023-05-16

**Authors:** M. Samper-Pardo, S. León-Herrera, B. Oliván-Blázquez, F. Méndez-López, M. Domínguez-García, R. Sánchez-Recio

**Affiliations:** 1 Institute for Health Research Aragon (IISAragon), Zaragoza, Spain; 2 Department of Psychology and Sociology, University of Zaragoza, Zaragoza, Spain; 3 Network for Research on Chronicity, Primary Care, and Health Promotion (RICAPPS), Barcelona, Spain; 4 Department of Preventive Medicine and Public Health, University of Zaragoza, Zaragoza, Spain

**Keywords:** Public health, Quality of life

## Abstract

The main objective of this study is to analyze the clinical efficacy of telerehabilitation in the recovery of Long COVID patients through ReCOVery APP for 3 months, administered in the Primary Health Care context. The second objective is to identify significant models associated with an improvement in the study variables. An open-label randomized clinical trial was conducted using two parallel groups of a total of 100 Long COVID patients. The first group follows the treatment as usual methods established by their general practitioner (control group) and the second follows the same methods and also uses ReCOVery APP (intervention group). After the intervention, no significant differences were found in favour of the group intervention. Regarding adherence, 25% of the participants made significant use of the APP. Linear regression model establishes that the time of use of ReCOVery APP predicts an improvement in physical function (b = 0.001; *p* = 0.005) and community social support (b = 0.004; *p* = 0.021). In addition, an increase in self-efficacy and health literacy also contribute to improving cognitive function (b = 0.346; *p* = 0.001) and reducing the number of symptoms (b = 0.226; *p* = 0.002), respectively. In conclusion, the significant use of ReCOVery APP can contribute to the recovery of Long COVID patients.

Trial Registration No.: ISRCTN91104012.

## Introduction

According to the World Health Organization (WHO)^1^, approximately 145 million people across the globe have been affected by Long COVID symptoms during the first 2 years of the pandemic. In the WHO’s European region, approximately 17 million people have been affected by this pathology, representing some 16% of the 102.4 million people infected by COVID-19 in this region and time period.

Given these data, the National Institute of Statistics has estimated that approximately 10–20% of infected people developed persistent symptoms more than twelve weeks following infection^2^. In 2020, and as a basis for future research, the UK's National Institute for Health and Care Excellence (NICE) distinguished between symptoms that continue four to twelve weeks after infection (ongoing symptomatic post-COVID-19, except for the loss of smell and taste) and those that persist for longer than twelve weeks (scientifically referred to as Long COVID)^3^. In October 2021, the WHO defined this new pathology as symptoms of a probable or confirmed SARS-CoV-2 infection that extend or develop 3 months after the initial infection, and that cannot be explained by an alternative diagnosis^4^. In this manuscript, this pathology is referred to as "Long COVID", a term that has been coined by the existing scientific evidence.

The underlying etiology of Long COVID, as well as its duration and degree of severity, remain limited. The study by Taquet et al.^5^ showed that, although persistent symptoms tend to be common with other viral infections, they appear to be more frequent with this particular infection. Previous research had suggested a higher prevalence of this disease in women (80%) as compared to men, mainly middle-aged women (between 48 and 58 years)^6^. More than two hundred symptoms of this pathology have been recorded, which may be persistent or cyclical over time^7^. According to systematic reviews, some of the most prevalent symptoms are fatigue or extreme tiredness, dyspnoea, myalgia, arthralgia, headache, cough, alteration to smell and taste, brain fog, lack of attention and concentration, as well as there being effects on mental health, among others^7–9^. These prolonged symptoms are disabling and affect the patient’s physical and mental health, altering their social, work, and family environment and their quality of life^10^.

It has been suggested that the management of Long COVID patients should be carried out and directed by general practitioners (GP)^11^. However, recommendations for outpatient medical care for these patients remain imprecise, and as such, GPs tend to rely on comparisons with similar conditions^12^. The great variability and fluctuation of symptoms make it difficult to provide suitable treatment^11^. A previous qualitative study verified the despair felt by Long COVID patients in Spain given the lack of improvement after different rehabilitation therapies^13^. Individualized rehabilitation and a global treatment plans are required, in a holistic sense of the disease, including, among others, local treatments, such as healthy lifestyles, physical rehabilitation, respiratory physiotherapy, cognitive rehabilitation, or psychological intervention^14^. However, the lack of knowledge to address this disease, in addition to the collapse of the health system, caused by the COVID-19 pandemic, has made it difficult to offer face-to-face supervised rehabilitative care, leading to the increased use of telerehabilitation (TR) strategies^15^.

A recent systematic review has verified that TR can improve dyspnea, functionality, and physical components of quality of life in COVID-19 and Long COVID patients^16^. Nevertheless, the meta-analysis by Seid et al.^17^ concludes that more studies are still required to investigate the effects of TR on the quality of life of these patients. The initial studies on the effectiveness of TR for Long COVID patients began to emerge in 2021, obtaining results that were beneficial for physical and respiratory functioning or quality of life, among others^18–21^, but they were observational, pilot studies or without a control group. These studies would serve as support for future large-scale investigations. It was in 2022 when the first clinical trials on TR for LONG COVID patients began to be published. The study by Li et al.^22^, with two parallel groups of one hundred and twenty previously hospitalized for COVID-19 with persistent dyspnea, designed a TR mobile application (APP), and improvements in physical performance were obtained in favor of the intervention group. Similarly, the study by Pehlivan et al.^23^ with thirty-four post-hospitalized COVID-19 patients, achieved significant improvements in dyspnea and physical functioning through TR. More recently, the study by Hajibashi et al.^24^ offered pulmonary TR combined with muscle relaxation for six weeks in post-hospitalized patients with COVID-19. Their results showed significant improvements for sleep quality or anxiety, but not for dyspnea or other variables related to physical functioning^24^. Finally, a 14-day TR program (strength and breathing exercises) carried out in Spain has proven effective in improving post-COVID-19 physical and respiratory function^25^ . All these studies suggest that TR may be a good option for the recovery of Long COVID patients. However, there seems to be controversy, given that some of them have included patients infected with COVID-19 less than 3 months ago, and cannot be considered Long COVID patients according to the previously mentioned WHO definition. In addition, the long-term effects of these types of interventions are unknown. For these reasons, it is still necessary to gain a better understanding of the TR treatments and rehabilitative processes that contribute to an individual’s recovery from Long COVID.

Due to all of the above, due to the increasing use of TR programs, the lack of treatment for these patients and the experimental studies carried out in other territories, it has been considered opportune to design and create a mobile APP called ReCOVery that serves as a TR for Long COVID patients. This APP is based on clinical guidelines for the management of patients with Long COVID and the needs identified by affected patients. ReCOVery has content that rehabilitates in order to improve the quality of life and strengthen the personal constructs (health literacy, activation and self-efficacy) of these patients, through their own rehabilitation and empowerment.

Hence, the main objective is to analyze the clinical efficacy of a TR through the ReCOVery APP, as compared to Treatment as Usual (TAU) for 3 months, administered in the Primary Health Care (PHC) context, as adjuvant treatment for individuals diagnosed with Long COVID. The second objective is to identify significant models associated with an improvement in the study variables.

## Methodology

### Study design

This study is an open-label randomized clinical trial (RCT) using two parallel groups of Long COVID patients. The first group follows the TAU methods established by the primary health care GP (control group) and the second follows the TAU methods and also uses the APP ReCOVery, as a coadjutant treatment in their recovery (intervention group). In addition, the intervention group attend three face-to-face sessions based on motivational methodology and APP management, with the aim of promoting adherence to the APP. Table 1 below shows the checklist for the description and replication of interventions (TIDieR).

**Table 1:** Template for intervention description and replication (TIDieR) checklist.

1. Brief name
Effectiveness of a telerehabilitation intervention using ReCOVery APP of Long COVID patients: A randomized, 3-month follow-up clinical trial
2. Why
Long COVID patients are suffering a great impact on their quality of life. However, the etiology of this pathology is still unknown. Different telerehabilitation strategies are being implemented to combat the varied and fluctuating symptoms. Hence, the main objective of this study is to analyze the efficacy of the ReCOVery mobile application over a period of 3 months. A second objective is to identify significant patterns associated with improvement
3. What (materials)
ReCOVery is a mobile application designed for this study and intended to offer rehabilitation treatment that improves the quality of life of Long COVID patients. Thus, the application was installed only on the personal devices of the participants in the intervention group
4. What (procedures)
First, the baseline assessments were performed face-to-face. Secondly, after randomization, the participants of the intervention group were summoned to three face-to-face sessions, the first two individual and the third group. From the first session until the next evaluation, the participants of the intervention group will have access to ReCOVery. Finally, after twelve weeks, all the participants are summoned again for a second evaluation
5. Who provided
A multidisciplinary approach was used, involving GPs, psychologists, physiotherapists, nurses, occupational therapists and social workers. All of them presented similar previous experiences in research, presented scientific knowledge about the pathology and, the corresponding ones, were taught to complete the selected scales and carry out the necessary interventions
6. How
The vast majority of the intervention was performed electronically, using ReCOVery. However, two individual sessions and one group session were held face-to-face at the beginning of the intervention. In addition, the evaluations were also carried out face-to-face. During the intervention, a contact telephone number was offered to all participants to notify any adverse event
7. Where
This project was developed in various primary care health centers in the territory of Aragon (Spain), both the recruitment and the evaluations
8. When and How Much
The intervention and, therefore, access to the APP began in March 2022 (baseline evaluation) and was available to participants in the intervention group until June 2022 (12-week evaluation). In that period of time they had access twenty-four hours a day, so they could use it freely
9. Tailoring
From the beginning it was planned that it should be the participants themselves who self-regulate. In this way, the contents were the same for everyone, but their intensity had to be controlled by themselves
10. Modifications
No modifications occurred to the planned intervention during the course of the study
11. How well (planned)
Adherence to the intervention protocol was good, given the number of dropouts was within the estimate. The reasons for abandonment were not going to the appointment or being reinfected with COVID-19 during the intervention
12. How well (actual)
The complete scheduled intervention program was delivered to both study groups (once the intervention was completed it was offered to the control group), without any deviation from the planned protocol

This RCT was registered in the ISRCTN Registry platform (Registry No.: ISRCTN91104012) on 10/02/2022. The original study protocol specifies multiple methodological aspects and has recently been published^26^.

### Recruitment of participants

The purposive sampling method was used to invite individuals to participate in the study. Potential study participants were PHC patients from the territory of Aragón (Spain), as well as interested parties from the "Long COVID Aragón" association for those affected, who were redirected to their GPs. When the GPs identified potential participants, they were provided with an information document and a form to verify that they met the criteria. Once the informed consent of the patient was obtained, the GPs notified the investigator so that he could contact the interested participants, subsequently reconfirming their inclusion or not according to the established criteria. Therefore, the settings in which the recruitment of potential participants was carried out were PHC offices.

The study population consisted of Long COVID patients (with a positive COVID-19 diagnostic test for longer than the previous twelve weeks and persistent symptoms) and adults (18 years or older). The exclusion criteria were: not having a positive COVID-19 diagnostic test for more than the previous twelve weeks; having a diagnosis of severe uncontrolled illness; significant risk of suicide; pregnancy and lactation; participation in a clinical trial over the past 6 months; existing structured rehabilitative or psychotherapeutic treatment by health professionals and the presence of any medical, psychological or social problems that may significantly interfere with the patient's participation in the study.

Recruitment was carried out consecutively until the estimated sample size was attained. The recruitment time was 3 months, from January to March 2022. A total of 100 PHC patients from the region of Aragon (Spain) were recruited.

### Sample size

To perform the estimated calculation of the necessary sample, the Spanish study by Dalbosco-Salas et al.^19^ was considered. Although the study is longitudinal, it presents a similar intervention that was also carried out in the PHC setting. To the best of our knowledge, no studies have been identified that consider Long COVID patients evaluating a similar intervention using a clinical trial methodology. Therefore, the pre-post score difference of the SF-36 instrument was used, considering the value of the highest possible standard deviation (SD) and a minimum expected difference of 19.3 points for the pre-post rating. A risk of 0.05 was accepted as well as a power of 95% in a two-sided contrast, and a maximum dropout rate of 10%. The minimum required sample size was 78 subjects.

Given the demand of the potentially interested participants, the researchers agreed to accept approximately 28% more participants, in accordance with the personal and material means available at that time. Therefore, the final sample size included 100 participants, 22 more than the required sample size.

### Randomization, assignment, and blinding of study groups

An independent researcher performed the individual randomization process using a computer-generated blind sequence with an alphabetically organized list of participants. Assignment to the intervention or control group was not blind, given the nature of the study. This researcher called each participant to confirm the assigned intervention and requested that they did not inform third parties of their assignment.

Participants in the control group were asked to continue with their current routines (complementary with exclusion criteria) and refrain from beginning any rehabilitation or similar activities that could affect this process. The intervention group was summoned in person and individually, and were asked to bring their personal mobile device with a charger to proceed with the installation of the APP and the start of the intervention.

### Development and evaluation of the APP and interventions

All participants continued with their TAU and were overseen by their PHC professionals and other medical specialists.

Participants assigned to the intervention group had access to the APP with rehabilitative content and attended three sessions on motivational methodology, APP management, and strengthening of their personal constructs (health literacy, self-efficacy, and personal activation). Two individual sessions and one group session were held over three consecutive weeks, after completing the baseline assessments and randomization process. The sessions were based on the motivational guide by Miller and Rollnick^27^, which intended to promote adherence to the APP. The individual sessions were guided by a clinical psychologist and lasted 20–30 min, during which the APP was installed and doubts regarding its use and management were resolved. It is important to consider that during the intervention ReCOVery APP was private, so that only the participants assigned to the intervention group had access to it from their personal phones, thus preventing possible leaks to the control group. The group sessions were held with a minimum of eight and a maximum of twelve participants and did not exceed one hour per session. These sessions were led by two clinical psychologists. All participants completed the sessions during the same weeks, with individual sessions being a prerequisite for group session attendance in the third week.

As for the ReCOVery APP architecture, a native APP was created with Java language, using the Android Studio platform. The design of a native APP was chosen, as opposed to a hybrid one, in order to make use of the device's own tools, such as notifications. This allowed the APP to remain updated. A human-centered design was selected. This technique aims to solve problems and needs by understanding the users themselves. Thus, the initial ReCOVery design was guided by symptoms, identified needs, and other information obtained through individual qualitative interviews and focus group discussions with patients diagnosed with long-term COVID-19^13^. Subsequently, the available scientific evidence was compiled using health recommendations and recovery exercises for patients with Long COVID. Different work subgroups were created in the multidisciplinary team to design and create content for each rehabilitation area. ReCOVery consists of six main modules, which need to be graduated and customized according to the specific needs and characteristics of each patient, as indicated in the previous instructions, thereby avoiding irreversible damage.

All of the details on the creation process, contents, and bibliographical references for the ReCOVery APP are detailed in the protocol article of this study^26^.

The main modules of the APP are:

1. Recommendations for adherence to the Mediterranean diet. Among others, it is recommended that potential nutritional deficiencies in vitamin D, vitamin B12, complex B, folic acid, and omega-3 fatty acids be supplied.
2. Recommendations to improve the quality of sleep and rest. The need to attain an average of 7 to 8 h of sleep each night is encouraged to ensure sufficient rest.
3. Physical exercise recommendations with graphic representations. The contents and instructions in this section were based on guidelines for the management of this disease or other pathologies with similar symptoms.
4. Respiratory physiotherapy exercises with video tutorial support.
5. Cognitive stimulation exercises with different difficulty levels. Three levels of cognitive stimulation exercises are provided, which are aimed at working on cognitive skills focused on executive function, difficulty maintaining attention, decreased processing speed, verbal fluency, and short-term memory deficits.
6. Participation in community resources. The aim is to promote participation in the local development process through different services, associations, or cultural activities, as well as groups affected by the same pathology.

### Follow-up of the intervention and adverse events

This was a remote and uncontrolled intervention. However, a follow-up call was made six weeks after the start of the intervention, that is, halfway through the process.

Prior to the start of the intervention, the researchers established the following adverse events: reinfection with COVID-19, use of emergency medical services, hospitalization or surgical interventions, or any other circumstance that could disrupt the intervention. In addition, all participants were provided with a telephone number to report any adverse events occurring throughout the study, either by phone call or text message. Participants were also asked about the occurrence of adverse events during the follow-up call and the second assessment. During the intervention, it was not necessary to assess adverse events other than those mentioned above. Two independent researchers, blind to the group assignment, assessed all reported adverse events (re-infections). Any disagreements were resolved through the participation of a third researcher.

### Main variable and measure

A total of two measurements were taken 3 months apart. These evaluations were carried out in person at a PHC Center in the participant’s city in order for them to be evaluated individually. The evaluations were carried out by two independent researchers with past experience in similar projects and actions. However, both researchers were instructed to evaluate using theoretical and practical sessions, avoiding biases in the process. A baseline evaluation (T0) was carried out prior to the start of the intervention, and a second effectiveness evaluation (T1) was carried out 3 months after the end of the first evaluation. Both evaluations were conducted over a period of two consecutive weeks. In addition, in the future, a new evaluation, which is not included in this article, will be made 6 months from the start of the intervention (T2).

The main variable is quality of life, which was assessed using the Short Form-36 Health Survey Questionnaire (SF-36)^28^. This questionnaire measures eight dimensions of health (vitality, physical functioning, bodily pain, general health perceptions, physical role functioning, emotional role functioning, social role functioning, and emotional wellbeing), which are grouped into two main components: physical health and mental health. Items are scored on five or six Likert scales ranging from one to three points, depending on the item type. The eight scales are scored from zero to one hundred, with scores above or below fifty indicating a better or worse health status, respectively. The Cronbach’s alpha obtained in this study was 0.84.

### Secondary variables

As for the secondary study variables, an ad hoc questionnaire was designed for the sociodemographic, clinical, and use of ReCOVery APP variables. In addition, a total of 10 validated scales were selected to further examine the Long COVID patient profiles. In all cases, validated Spanish adaptations of the original scale were used.

- The following sociodemographic variables were studied: gender (man, woman, other), age, civil status (married or in a couple/single, separated, divorced, or widowed), education (no studies or primary studies/ secondary or university studies), and occupation (employee, unemployed, employee with temporary work disability (TWD), retired, others).
- The clinical variables related to post-COVID-19 that were studied were time since infection (months) and the number of self-reported persistent symptoms at the time of each evaluation, using a list of thirty persistent symptoms typical of Long COVID patients according to past literature^7–9,29^.
- The use of ReCOVery APP variables were: time of APP use during the 3 months, expressed in minutes. As for adherence to the APP, significant use was estimated as being fifteen minutes a day, for five days a week, for twelve weeks (one thousand two hundred minutes or more).
- Cognitive domains, such as memory, attention, language, or working memory, were assessed using the Montreal Cognitive Assessment (MoCA) questionnaire^30^. This has a total score of thirty points with the cut-off point for the detection of mild cognitive impairment being less than twenty-six points. The Cronbach's alpha obtained in this study was 0.457.
- The physical functioning variable was measured using the thirty-second Sit-to-Stand Test^31^. This test assesses endurance at high power as well as speed in terms of muscular endurance or strength and records the number of times an individual can stand up and sit down completely. It has good test–retest reliability (0.84 < R < 0.92).
- Affective status, in relation to depression and anxiety disorders, was measured with the Hospital Anxiety and Depression Scale (HADS) questionnaire^32^. The HADS contains fourteen items, each of which corresponds to a four-point Likert-type scale (zero to three), with scores ranging from zero to forty-one for its total score. Higher scores indicate more severe symptoms. The Cronbach's alpha obtained in this study was 0.91.
- Sleep quality was measured using the Insomnia Severity Index (ISI) questionnaire^33^. This self-report scale has seven items, with each response ranging from zero to four, and an overall score ranging from zero to twenty-eight, with a higher score indicating a greater severity of insomnia. The Cronbach's alpha obtained in this study was 0.86.
- Social Support was measured using the Medical Outcomes Study Social Support Survey (MOS-SS) questionnaire^34^. This is a self-report instrument consisting of four subscales (emotional/informational, tangible, affectionate, and positive social interaction) and an overall functional social support index. It has nineteen items and uses a five-point Likert scale. Higher scores indicate more support. The Cronbach's alpha obtained in this study was 0.94.
- Community social support was measured using the Perceived Community Support Questionnaire (PCSQ)^35^. This consists of twenty-five Likert-type items with a scale from one to five evaluating: community integration, community participation, social support of informal systems, and social support of formal systems. Higher scores suggest more community social support. The Cronbach's alpha obtained in this study was 0.49.
- Regular physical activity levels were measured using the International Physical Activity Questionnaire-Short Form (IPAQ-SF)^36^. This has seven items and records activity at four intensity levels. The number of minutes walked score was used in the analysis of this study.
- The following personal factors related to behavior were collected:
  - Self-efficacy, measured with the Self-Efficacy Scale-12 (GSES-12)^37^. This scale contains twelve items with a Likert scale from one to five. The resulting score ranges between twelve and sixty. Higher scores indicate greater self-efficacy. The Cronbach's alpha obtained in this study was 0.76.
  - Patient activation in their own health was measured using the Patient Activation Measure (PAM) questionnaire regarding the management of their health^38^. This questionnaire contains thirteen items with a Likert scale from one (strongly disagree) to four (strongly agree). The resulting score ranges between thirteen and fifty-two. Higher scores indicate better activation. The Cronbach's alpha obtained in this study was 0.87.
  - Health literacy was measured using the Health Literacy Europe Questionnaire (HLS-EUQ16)^39^. This questionnaire contains sixteen items, ranging from 1 to 4. The resulting score ranges between sixteen and sixty-four. Higher scores indicate poorer health literacy. The Cronbach's alpha obtained in this study was 0.87.

#### Statistical analysis

Firstly, a descriptive analysis of all of the variables was carried out, using frequencies and percentages for categorical variables and means and standard deviation for continuous variables. A between-groups comparison was developed after randomization according to the study variables, with chi-square being used for categorical variables and Student T for continuous variables.

To analyze the APP’s effectiveness, a per-protocol analysis was performed, comparing baseline, 3 months, and the 3-month-baseline differences between both groups using Student T. To analyze the variables associated with effectiveness, a linear regression was performed considering the difference in the score at 3 months and at baseline for each of the variables as the dependent variable. The independent variables of age, gender, minutes of APP use, increase in self-efficacy, health literacy, and patient activation were introduced into the model.

#### Ethical questions

Ethical approval was granted by the Clinical Research Ethics Committee of Aragon (PI21/454). The procedures followed during the creation of this work complied with the ethical standards of the previously mentioned committee and with the Declaration of Helsinki of 1975. All of the subjects signed an informed consent form. Their data were anonymized and will only be used for study purposes. The ethics committee will be notified of any relevant modifications.

#### Ethical approval and consent to participate

This study received the approval of the Ethics Committee for Clinical Research of Aragon, Spain. All procedures were carried out in accordance with the ethical standards of this Committee. Written informed consent was obtained from all study participants.

## Results

**Figure 1:**
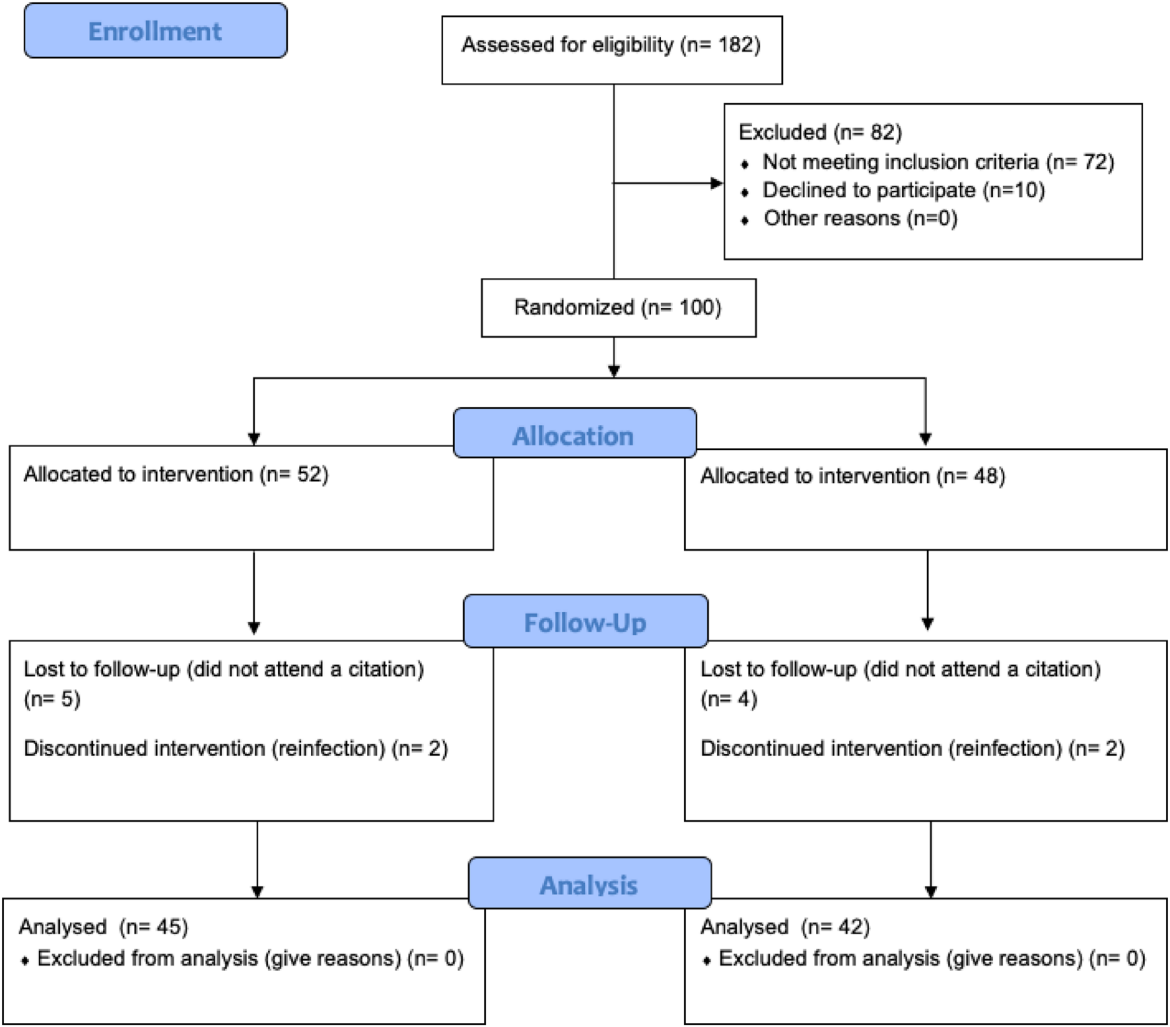
CONSORT Flowchart.

Initially, a total of 182 participants were evaluated for eligibility, of which 82 (45.05%) did not participate. As reflected in Fig. 1, 72 participants did not meet the inclusion criteria and 10 did not participate due to a lack of interest. Ultimately, 100 participants were included and randomized, 52 in the intervention group and 48 in the control group. The 3-month evaluation was completed by 87 participants, 45 of which belonged to the intervention group and 42 to the control group. A total of 13 participants were excluded from the 3-month analyses, 4 due to reinfection and 9 for their failure to attend a face-to-face session or the evaluation session.

Firstly, as seen in Table 1, the descriptive analysis revealed that of the 100 participants, 80 were female and 20 were male. The participants had a mean of 16.47 (SD: 5.99) persistent symptoms, low quality of life (both physical and mental health) and low physical and mental functioning (reflected in the scores from the MoCa and Sit-to-Stand Test), and high scores on depression and anxiety, social support, and self-efficacy perception. In Table 2, a comparison between the intervention and control groups is also shown. This analysis subsequently revealed no significant differences between groups.

**Table 2:** Description of sociodemographic and clinical variables and comparations of intervention-control groups.

Variables	Total sample N = 100	Intervention group N = 52	Control group N = 48	*p*-value
*Gender (%)				
Men	20 (20%)	8 (15.4%)	12	0.230
Women	80 (80%)	44 (84.5%)	33	
Age	48.28 (9.26)	48.25 (10.36)	48.31 (8.01)	0.963
Perceived age	58.10 (14.69)	59.69 (15.02)	56.38 (14.27)	0.260
*Marital status (%)				
Married or in a couple	70 (70%)	35 (67.3%)	35 (72.9%)	0.541
Single, separated, widowed	30 (30%)	17 (32.7%)	13 (27.1%)	
*Educational level (%)				
Without studies or primary studies	9 (9%)	5 (9.6%)	4 (8.3%)	0.823
Secondary or university studies	91 (91%)	47 (94.4%)	44 (91.7%)	
*Employment (%)				
Employee	46 (46%)	20 (38.5%)	26 (54.2%)	0.350
Unemployed	5 (5%)	4 (7.7%)	1 (2.1%)	
TWD	37 (37%)	21 (40.4%)	16 (33.3%)	
Retired	9 (9%)	6 (11.5%)	3 (6.3%)	
Others	3 (3%)	1 (1.9%)	2 (4.2%)	
Time since the contagious	16.12 (6.34)	15.75 (6,56)	16.52 (6.14)	0.545
Number of persistent symptoms	16.47 (5.99)	17.50 (5.29)	15.35 (6.55)	0.074
SF-36				
SF-36 Physical health	32.19 (16.61)	29.06 (13.67)	35.58 (18.86)	0.053
SF-36 Mental health	34.77(19.31)	32.64 (17.98)	37.09 (20.59)	0.254
Montreal cognitive assessment	23.64 (3.85)	23.48 (4.20)	23.81 (3.46)	0.667
Sit-to-Stand test	10.37 (3.49)	9.87 (3.77)	10.92 (3.10)	0.131
Affective state (HADS)	17.61 (8.31)	17.86 (7.98)	17.33 (8.74)	0.752
Insomnia severity index	11.34 (6.58)	11.13 (7.21)	11.56 (5.89)	0.745
Social support (MOS-SS)	83.84 (16.33)	83.42 (16.22)	84.29 (16.61)	0.792
Community social support	82.06 (14.29)	82.33 (15.51)	81.7 (12.99)	0.846
Self-efficacy	44.66 (7.51)	44.57 (6.49)	44.75 (8.55)	0.910
Patient activation	39.82 (6.16)	38.92 (7.24)	40.79 (4.61)	0.210
Health literacy	32.10 (7.03)	32.94 (7.84)	31.18 (5.98)	0.125

Statistics used: Mean and standard deviation except for variables with *, for which frequencies and percentages have been used. For comparation, T student except form variables with *, for which chi-squared has been used. *TWD* Temporary work disability, *HADS* Hospital anxiety and depression scale, *MOS-SS* Medical outcomes study social support survey (MOS-SS).

As seen in Table 3, the analysis comparing the pre-intervention and 3-month post-intervention data, and considering the raw scores of both groups, a significant decrease was found in the number of persistent symptoms, with there being less in the control group (*p*-value: 0.09; CI:  − 0.44–5.41). There were no differences between groups for the other variables.

**Table 3:** Outcome data at baseline and 3-month follow-up.

Variables	Intervention group N = 52 mean (SD)	Control group N = 48 mean (SD)	Significance *p*-value (CI)
Primary outcomes			
SF-36 Physical Health			
Baseline (T0)	29.06 (13.67)	35.58 (18.86)	0.053 (− 13.12; 0.07)
3 months (T1)	33.80 (12.19	42.30 (20.31)	0.021 (− 16.30; 1.40)
T1–T0	4.56 (12.14)	8.02 (14.38)	0.234 (− 9.20; 2.28)
SF-36 Mental health			
Baseline (T0)	32.64 (17.98)	37.09 (20.59)	0.252 (− 12.16; 3.24)
3 months (T1)	37.35 (20.01)	40.29 (19.59)	0.491 (− 11.38; 5.50)
T1–T0	5.07 (16.10)	3.20 (18.27)	0.615 (− 5.49; 9.23)
Secondary outcomes			
Number of persistent symptoms	17.50 (5.29)	15.35 (6.55)	0.074 (− 0.23; 4.52)
Baseline (T0)	16.48 (4.65)	14.00 (6.64)	
3 months (T1)	− 0.27 (3.03)	− 1.43 (3.79)	0.09 (− 0.44; 5.41)
T1–T0			0.188 (− 0.58; 2.90)
Montreal cognitive assessment			
Baseline (T0)	23.48 (4.20)	23.81 (3.46)	0.667 (− 1.85; 1.19)
3 months (T1)	24.13 (4.45)	24.14 (3.84)	0.991 (− 1.78; 1.76)
T1–T0	0.91 (4.24)	0.30 (2.87)	0.439 (− 0.93; 2.14)
Si-to-Stand Test			
Baseline (T0)	9.87 (3.77)	10.92 (3.10)	0.131 (− 2.42; 0.31)
3 months (T1)	10.65 (3.66)	11.28 (3.89)	0.462 (− 2.30; 1.05)
T1–T0	0.32 (2.24)	− 0.28 (4.84)	0.806 (− 1.36; 1.06)
Affective state			
Baseline (T0)	17.86 (7.98)	17.33 (8.74)	0.752 (− 2.80; 3.86)
3 months (T1)	17.20 (8.72)	16.00 (9.95)	0.553 (− 2.80; 5.20)
T1–T0	− 0.28 (4.84)	− 1.21 (6.17)	0.441 (− 1.45; 3.30)
Insomnia severity index			
Baseline (T0)	11.13 (7.21)	11,56 (5,89)	0.745 (− 3.03; 2.17)
3 months (T1)	10.50 (5.53)	10,33 (5,94)	0.893 (− 2.30; 2.63)
T1–T0	− 0.54 (5.35)	− 1,47 (5,94)	0.449 (− 1.50; 3.36)
Social support			
Baseline (T0)	83.42 (16,22)	84.29 (16.61)	0.792 (− 7.39; 5.65)
3 months (T1)	82.82 (17,32)	82.26 (16.59)	0.878 (− 6.67; 7.79)
T1–T0	− 0.15 (12.03)	− 1.09 (8.60)	0.675 (− 3.50; 5.38)
Community social support			
Baseline (T0)	82.33 (15.51)	81.7 (12.99)	0.846 (− 5.10; 6.22)
3 months (T1)	84.53 (20.53)	79.92 (12.87)	0.211 (− 2.66; 11.87)
T1–T0	2.42 (18.5)	− 2.45 (12.22)	0.153 (− 1.84; 11.59)
Self-efficacy			
Baseline (T0)	44.57 (6.49)	44.75 (8.55)	0.910 (− 3.21; 2.86)
3 months (T1)	43.31 (9.10)	44.92 (8.69)	0.399 (− 5.41; 2.17)
T1–T0	− 1.00 (6.59)	0.28 (5.80)	0.336 (− 3.92; 1.35)
Patient activation			
Baseline (T0)	38.92 (7.24)	40,79 (4.61)	0.210 (− 4.26; 0.52)
3 months (T1)	40.24 (6.90)	39,92 (5.72)	0.816 (− 2.39; 3.02)
T1–T0	0.91 (8.44)	− 1.36 (5.32)	0.136 (− 0.72; 5.28)
Health literacy			
Baseline (T0)	32.94 (7.84)	31.18 (5.98)	0.125 (− 1.00; 4.51)
3 months (T1)	32.00 (7.35)	30.32 (7.16)	0.291 (− 1.46; 4.81)
T1–T0	− 0.53 (6.89)	− 0.05 (7.54)	0.760 (− 3.61; 2.65)

Upon analyzing the APP use in the intervention group, the range of use during the 3 months oscillated between 10.95 min and 5,764.81 min. The mean amount of use was 835.52 min (SD: 1090.57) during the 3 months. Only 17 participants (25%) from the intervention group engaged in significant use of the APP (with significant use being considered as being at least 15 min per day, for 5 days per week, for 12 weeks). This data suggests a poor adherence to the mobile APP.

As for the variables associated with an improvement in the analyzed variables, the multivariate models revealed that there were no significant models related to the effectiveness in the quality of life (SF-36 physical and mental health), affective state, sleep quality, and social support. However, significant models did show a decrease in the number of persistent symptoms, and an improvement in cognitive functioning (MoCA), physical functioning (Sit-to-Stand Test), and Community social support (PCSQ). As Table 4 shows, the decrease in persistent symptoms was predicted by an increase in health literacy, explaining 19.2% of the variance. The improvement in cognitive functioning was predicted by an increase in the self-efficacy construct, which explains 36.5% of the variance. The improvement in physical functioning was predicted by the minutes of APP use and being a man. This model explains 28.7% of the variance. Finally, improvement in community social support was predicted by the minutes of APP use and an increase in health literacy. This model explains 19.8% of the variance.

**Table 4:** Linear regression model with regard to improvements in the number of persistent symptoms, cognitive functioning (MoCA), physical functioning (Sit-to-Stand Test), and Community social support (PCSQ).

Decrease in the number of persistent symptoms	Coefficient	*p* value	CI below 95%	CI above 95%
Increase in health literacy	0.226	0.002	0.087	0.365
R^2^	0.192			
R^2^_adj_	0.102			

Cognitive functioning (MoCA)	Coefficient	*p* value	CI below 95%	CI above 95%
Increase in self-efficacy	0.346	0.001	− 0.538	− 0.154
R^2^	0.365			
R^2^_adj_	0.265			

Physical functioning (Sit-to-Stand Test)	Coefficient	*p* value	CI below 95%	CI above 95%
Gender	− 2.454	0.016	0.928	8.732
Minutes of APP use	0.001	0.005	0.000	0.002
R2	0.287			
R2adj	0.226			

Community social support (PCSQ)	Coefficient	*p* value	CI below 95%	CI above 95%
Increase in self-efficacy	0.634	0.036	0.042	1.226
Minutes of APP use	0.004	0.021	0.001	0.008
R2	0.198			
R2adj	0.135			

## Discussion

The findings of this study indicate that the use of ReCOVery APP for 3 months was not significantly more effective in producing an improvement in the quality of life of Long COVID patients. Mostly, the participants did not significantly use the APP nor allowed it to be an effective tool. However, linear regressions model identified significant models of improvement predicted by time of ReCOVery APP use, increased self-efficacy, increased health literacy, and male gender. These evidences with adequate adherence could achieve significant improvements and encourage new management guidelines based on this evidence.

Our effectiveness analyses revealed more improvement in the intervention group in terms of mental health (SF-36), cognitive state (MoCA), physical state (Sit-to-Stand), community social support (PCSQ), and patient activation (PAM), although without attaining significant improvement as compared to the control group. The great impact suffered by these patients suggests that different RCTs have already been implemented and have evaluated the effectiveness of a rehabilitative intervention on the quality of life of post-COVID-19 patients^40–42^. Contrary to our study, some studies did achieve significant improvements in the quality of life of COVID-19 patients. The RCT by Nambi et al.^41^ identified significant improvements in the physical component of the SF-12 scale for the low-intensity versus high-intensity aerobic activity group^40^; like Liu et al.^40^, finding improvements in all vital dimensions of the SF-36 after six weeks of respiratory rehabilitation training^41^. Moreover, the pilot study by Abodonya et al.^43^, based on the effects of inspiratory muscle training for 2 weeks, verified a significant improvement in quality of life in favor of the intervention group according to the EQ-5D-3L questionnaire^42^. These studies, however, have relied upon a small number of participants^40,42^ or have certain potential limitations with regard to internal validity^41^. Moreover, it is crucial to highlight that many of these RCTs, according to their inclusion criteria, were carried out with post-COVID-19 patients, but without persistent symptomatology for 3 months or more. In this way, it is necessary to differentiate and emphasize that they would not be Long COVID patients, as in our study. In this sense, Long COVID patients may present greater deterioration, given that the initial period of rehabilitation is essential^43^. In fact, depending on the patient's condition, some interventions can cause important damage^43^, as seen in the RCT by Mohamed and Alawna^45^, in which an intervention based on aerobic activity led to a decrease the quality of life of post-COVID-19 patients^44^. Therefore, it should be considered that the chronic symptoms of Long COVID patients may require a longer rehabilitation period, greater than twelve weeks.

Thus, TR in post-COVID-19 patients (possibly in the first weeks after infection) is feasible to improve their quality of life^45^. However, large-scale studies are still needed with patients with persistent symptoms for at least least twelve weeks after infection. In fact, a case report by Mayer et al.^47^ a Long COVID patient participated in biweekly physiotherapy sessions for eight weeks, achieving improvements in some physical variables studied, but not their quality of life^46^. Regarding TR, A systematic review states that TR appears to be useful for Long COVID patients. However, this study warns that a subgroup of patients presents adverse effects (episodes of dizziness)^45^. In this same line, the study by Vieira et al.^16^ suggests investigating mixed models of classic rehabilitation and TR, with face-to-face and remote elements, so that trained professionals can adjust and/or stop the activity at the most precise moments. As background to this reality, it is worth noting the study of Lau et al.^48^, in which a physical training intervention was used for the recovery of patients infected by SARS-CoV in 2002. The intervention did not offer a significant improvement in the quality of life of the participants^47^.

*However, the non-significant improvements in this study make it necessary to consider adherence to ReCOVery APP.* The adherence to the APP was low, with only 25% of the participants in the intervention group engaging in significant APP use during the twelve weeks. It is not known if APP adherence decreased over the weeks, potentially leading to an improvement over the short term, but not over the medium term, as identified by some recently mentioned studies. Furthermore, a recent study by Deng et al.^49^ verifies that treatment adherence is a common problem for people with mental disorders, such as depression or anxiety, which may occur with Long COVID patients, given the negative impact on their emotional well-being, as verified by our analyses (mental Health SF-36) and previous evidence^48^. Rather, a systematic review of post-COVID-19 TR states that TR can increase patient adherence as compared to face-to-face rehabilitation, given its convenience and accessibility^16^. In addition, it is mentioned that daily communication via a software platform or reminders would increase participant adherence. These statements are inconsistent with the results of this study. The low adherence of our results remains a mystery that could possibly be resolved by future qualitative research on our participants.

Moreover, our linear regressions model identified significant models that explained the improvement in cognitive functioning (Mocha), physical functioning (Sit-to-Stand Test), and community social support (PCSQ), as well as the decreased number of symptoms, in relation to the time of APP use and the improvement of other secondary study variables. After reviewing the scientific evidence, these results are analyzed below in relation to evidence on COVID-19 and evidence with other pathologies.

An increase in health literacy predicts a decrease in the number of persistent symptoms. As noted by Liu et al.^42^, health literacy refers not only to the knowledge of health and care of the health system, it is defined as an individual’s ability to obtain and process knowledge and information to maintain and improve health through self-management in collaboration with health providers^49^. A European survey has verified that health literacy has a positive impact on patients with chronic diseases, especially those having a lower level of education or health knowledge^39^. In this way, the participants who have increased their health literacy may have self-managed their persistent symptoms better, possibly making better use of health services and thus reducing said symptoms. In turn, the increase in health literacy and greater use of the ReCOVery APP are predictors of greater community social support. A recent systematic review states that associations between self-reported health literacy and medication adherence are quite consistent^50^, so in this intervention, better health literacy and increased APP use would make people follow the recommendations to begin rehabilitation processes in the community, in addition to attending associations for affected people. According to this idea and with regard to the COVID-19 pandemic, low levels of health literacy are related to the ability to assess and trust online health information^51^, so an increase in knowledge of the management of Long COVID in terms of risks, contagion, as well as positive psychological repercussions, could have encouraged the integration of these patients in community services. In fact, a meta-analysis relating literacy on the measures resulting from the COVID-19 pandemic indicates that people with low health literacy revealed a greater tendency to accept misinformation about COVID-19 circulating over social media platforms and social networks, as well as decision-making related to health^52^.

Furthermore, improved cognitive functioning is predicted by an increase in the self-efficacy construct (GSES-12). As with our results, a prospective multicenter observational study by Jongen et al.^54^ concluded that self-efficacy can positively affect the cognitive performance of patients with multiple sclerosis^53^. In fact, self-efficacy has the potential to reduce cognitive stressors^54^. A cross-sectional study of patients with cerebral palsy related to increased self-efficacy with improved quality of life, both mental and physical, through the use of the GESES-12 and SF-36 questionnaires^55^.

Finally, improved physical functioning (Sit-to-Stand) is predicted by the minutes of APP use and by being a man. The intervention group’s baseline score on the Sit-to-Stand Test improved, whereas that of the control group worsened. A systematic review including RCTs with patients having post-COVID-19 sequelae based on physical rehabilitators also verified the improvement of these patients on the 30-s Sit-to-Stand scale^56^. More specifically, the study by De Souza et al.^58^, based on low-intensity pulmonary rehabilitation for COVID-19 survivors, saw improvements in this test, in addition to their daily physical activity and fatigue^57^. For this reason, rehabilitation exercises, both physical and respiratory, as well as the daily APP recommendations have led to improvements in this area. Moreover, previous evidence supports the idea that men with chronic diseases have a greater genetic predisposition than women to improve their physical functioning^58^, given the greater bone and muscle wear and tear suffered by women with aging^59^. Therefore, improvements in physical functioning would be expected to be greater in the participating men.

In terms of study limitations, first, the development of the intervention overlapped with periods in which COVID-19 infections were on the rise, affecting PHC care in the region. Second, the variables collected are based on the self-perceptions of the participants; therefore, we must trust their statements, even if it is not possible to objectively verify them. Third, the symptoms themselves cause not only physical but also mental limitations, to starting and following rehabilitative interventions. Finally, due to the nature of the intervention, all participants were informed of the assigned intervention during the RCT. There are also various strengths to the study. To the best of our knowledge, it is the first RCT conducted exclusively on Long COVID patients, challenging the chronological order of symptoms and obtaining scientific evidence to support future research with this group of patients. In addition, a specific APP has been designed and used for this study.

Future RCTs are required to assess the efficacy of TR-based interventions on Long COVID patients. As for APP use, mixed-method studies should be carried out to investigate the specific causes of poor adherence to the APP and to determine how to improve adherence and compliance rates. These studies are necessary to identify new significant models that contribute to improving the quality of life and symptoms of these patients, while also promoting evidence on their clinical management to support PHC professionals.

## Data Availability

The datasets used and/or analyzed during this study are available from the corresponding author upon reasonable request.
